# Draft genome sequence, annotation, and SSR mining data of *Elaeidobius kamerunicus* Faust., an essential oil palm pollinating weevil

**DOI:** 10.1016/j.dib.2021.106745

**Published:** 2021-01-18

**Authors:** Ardha Apriyanto, Van Basten Tambunan

**Affiliations:** a Research and Development, PT. Astra Agro Lestari Tbk, Jl. Puloayang Raya Blok OR I, Kawasan Industri Pulogadung, Jakarta Timur, Indonesia; b Biopolymer Analytics, Institute of Biochemistry and Biology, University of Potsdam, Karl-Liebknecht-Str. 24-25, Building 20, Potsdam-Golm, Germany

**Keywords:** Whole-genome sequencing, NGS, Simple Sequence Repeat, Weevil, Curculionidae, Oil Palm, Pollinator, Genomics

## Abstract

*Elaeidobius kamerunicus* Faust. (Coleoptera: Curculionidae) is an essential insect pollinator in oil palm plantations. Recently, researches have been undertaken to improve pollination efficiency using this species. A fundamental understanding of the genes related to this pollinator behavior is necessary to achieve this goal. Here, we present the draft genome sequence, annotation, and simple sequence repeat (SSR) marker data for this pollinator. In total, 34.97 Gb of sequence data from one male individual (monoisolate) were obtained using Illumina short-read platform NextSeq 500. The draft genome assembly was found to be 269.79 Mb and about 59.9% of completeness based on Benchmarking Universal Single-Copy Orthologs (BUSCO) assessment. Functional gene annotation predicted about 26.566 genes. Also, a total of 281.668 putative SSR markers were identified. This draft genome sequence is a valuable resource for understanding the population genetics, phylogenetics, dispersal patterns, and behavior of this species.

## Specifications Table

**Table:** 

Subject	Omics: Genomics
Specific subject area	Insects, Coleoptera, Oil Palm, Weevil, Whole-genome sequencing (WGS)
Type of data	Table
	Figure
	Raw DNA sequencing reads
	Draft genome assembly
	Repeat elements file
	Simple sequence repeat file
	Genome annotation file
How data were acquired	Paired-end sequencing on Illumina Nextseq 500 platform.
Data format	Raw – Fastq
	Analyzed – Fasta, gff
Parameters for data collection	DNA from one male adult individual (monoisolate) was used.
Description of data collection	DNA from the whole-body was extracted. DNA purity and concentration were measured before sequencing. DNA sequences obtained by Illumina Nextseq 500 platform followed by de novo assembly using SPAdes.
Data source location	Institution: Research and Development, PT. Astra Agro Lestari Tbk
	City/Town/Region: Pangkalan Lada, Kalimantan Tengah
	Country: Indonesia
	Latitude and longitude for collected samples/data:
	(2°25′28.6″S, 111°47′26.8″E).
Data accessibility	All data in this article are available at NCBI, BioProject number PRJNA637822.
	Direct URL to data: https://www.ncbi.nlm.nih.gov/bioproject/PRJNA637822
	Whole-genome sequence data are accessible at NCBI under GenBank accession number JACGEL000000000
	Direct URL to data: https://www.ncbi.nlm.nih.gov/nuccore/JACGEL000000000.1
	The raw sequence data with this article are accessible under SRA accession number SRR12726955-SRR12726958.
	Direct URL to data:
	https://www.ncbi.nlm.nih.gov/sra/?term=SRR12726955
	https://www.ncbi.nlm.nih.gov/sra/?term=SRR12726956
	https://www.ncbi.nlm.nih.gov/sra/?term=SRR12726957
	https://www.ncbi.nlm.nih.gov/sra/?term=SRR12726958
Related research article	A. Apriyanto, V.B. Tambunan, The complete mitochondrial genome of oil palm pollinating weevil, *Elaeidobius kamerunicus* Faust. (Coleoptera: Curculionidae), Mitochondrial DNA Part B. 5(3) (2020) 3450–3452. https://doi.org/10.1080/23802359.2020.1823899

## Value of the Data

• This article provides the draft genome sequence data of *Elaeidobius kamerunicus* Faust. (Coleoptera: Curculionidae) and thus addresses a knowledge gap of genome sequence within the order Coleoptera.
• The draft genome sequence of this species will be useful for entomologists interested in functional genomics, population genetics, phylogenetics, and selection by breeding.
• This dataset can be used as a reference for future complete genome assembly of this species.
• The newly developed SSR markers dataset in this report should be useful tools for assessing the genetic diversity, conservation, and bio management of this species.

## 1 Data Description

*Elaeidobius kamerunicus* Faust. (Coleoptera: Curculionidae) is an essential insect pollinator in oil palm plantations. This species is native to the tropical Africa region but introduced into Asia, including Indonesia [1]. The introduction of this weevil species into oil palm plantations successfully improved fruit set, increased the yield of oil palm, and reducing the need for assisted pollination [2]. Recent studies of this species have only focused on analyzing genetic diversity and species identification [1], [3], [4]. Interestingly, several divergent mitochondrial lineages in this species have been discovered based on the information of cytochrome c oxidase subunit I (COI) and cytochrome c oxidase subunit II (COII) gene sequences [1], [3]. Our recent study successfully obtained the complete mitochondrial genome of *E.kamerunicus* from the partial dataset from this report, representing the first complete mitogenome for this species [5]. Nevertheless, the genomic resources of *E.kamerunicus* remain underdeveloped compared with many other agricultural insect species.

This article presents the first draft genome assembly, annotation, and SSR marker data of the oil palm pollinating weevil, *Elaeidobius kamerunicus* Faust. (Coleoptera: Curculionidae). All raw sequencing reads data (34.97 Gb) used for genome assembly were deposited in the NCBI Short Read Archive (SRA) database. All of these SRA data are retrievable under the accession number SRR12726955-SRR12726958.

The assembled draft genome was constructed using the filtered reads, which is about 73.71% of the total raw sequence reads. The final draft genome assembly was 269.79 Mb containing 364.527 scaffolds with 31.71% GC (Table 1). The genome project information has been deposited in the NCBI GenBank under the Bioproject ID: PRJNA637822. The whole-genome sequencing (WGS) data can be retrieved from the NCBI GenBank under accession JACGEL000000000.

**Table 1:** Draft genome assembly statistic of *E.kamerunicus*.

Statistics	JACGEL000000000
Number of scaffolds	364,527
Number of scaffolds (>= 0 bp)	364,527
Number of scaffolds (>= 1000 bp)	82,506
Largest scaffolds (bp)	16,904
Total length (bp)	269,798,182
Total length (>= 0 bp)	269,798,182
Total length (>= 1000 bp)	145,163,070
N50	1084
N75	568
L50	72,645
L75	157,429
GC (%)	31.71

The assembled *E.kamerunicus* genome analyzed with BUSCO tools showed 59.9% completeness, indicating the genome to be of good quality. We found about 638 complete orthologs genes (C: 59.9%), 632 orthologs complete genes and single-copy (S: 59.3%), 6 orthologs complete genes and duplicated (D: 0.6%), 323 orthologs fragmented genes (F: 30.3%) and 105 missing genes (M: 9.8%).

**Fig. 1:**
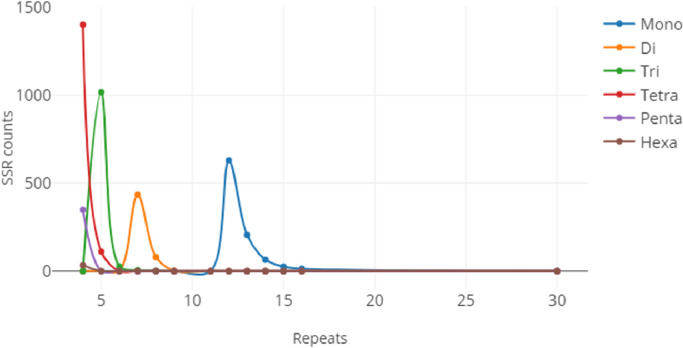
Perfect SSR distribution for each SSR type based on the number of repeats.

**Fig. 2:**
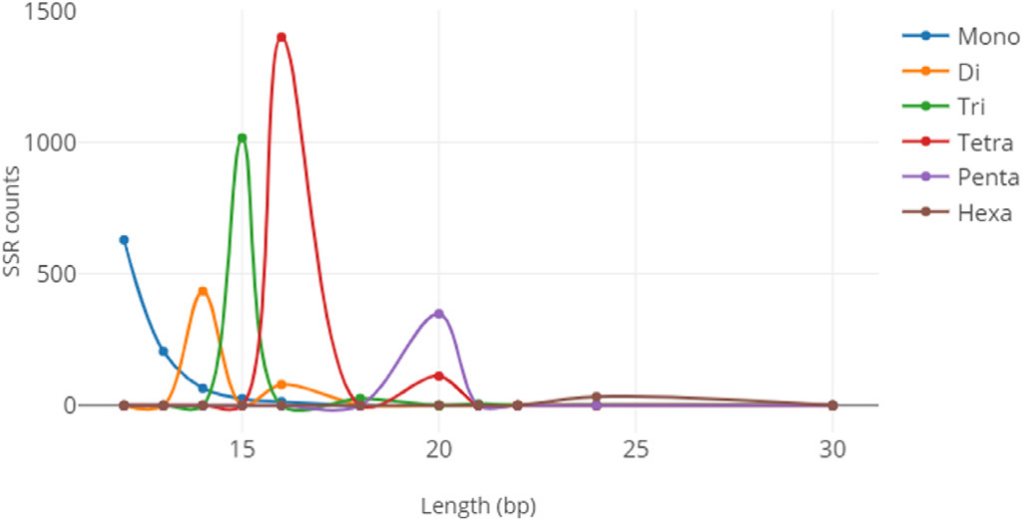
Perfect SSR distribution for each SSR type based on sequence length (bp).

The assembled draft genome of *E.kamerunicus* was used to identify simple sequence repeat (SSR) or microsatellite markers. In this dataset, we reported about 4.396 perfect SSRs (pSSRs), 3 compound SSRs (cSSRs), 251.377 imperfect SSRs (iSSRs), and 25.892 variable number tandem repeats (VNTRs) inside the *E.kamerunicus* genome. The annotation files of pSSRs, cSSRs, iSSRs, and VNTRs are provided in Supplementary file S1-S4, respectively. The distribution of perfect SSRs (pSSRs) based on their motif and sequence length can be seen in Fig. 1, Fig. 2, respectively.

**Fig. 3:**
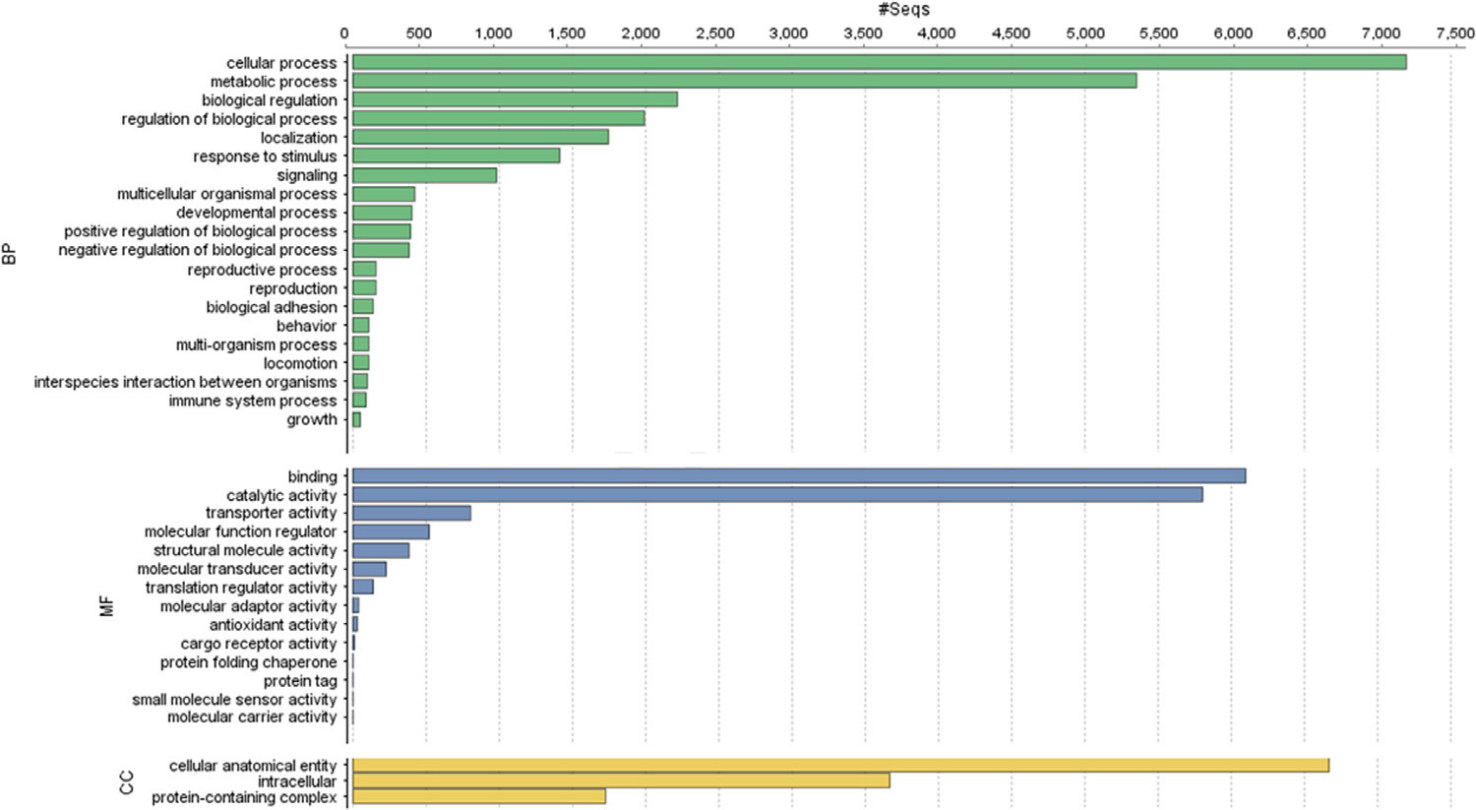
Histogram representing the gene ontology distribution of the annotated *E.kamerunicus* genes. The functionally annotated genes were assigned to three main GO categories: Biological Process (BP), Molecular Function (MF), and Cellular Component (CC).

Table 2 provides the detailed information of repetitive elements detected in this assembled genome. The annotation data of repetitive elements can be found in Supplementary file S5. Functional gene annotation pipeline predicted about 26.566 genes, 14.145 were found to have GO term assigned to them. The GO term classification and distribution can be seen in Fig. 3. The genome annotation data can be found in Supplementary file S6.

**Table 2:** Summary of repetitive elements in the assembled genome of *E.kamerunicus.* Most repeats fragmented by insertions or deletions have been counted as one element.

Repeat class/family	Number of elements	Length occupied
**SINEs**	9	630
Penelope	15	1110
**LINEs**	402	25,989
L2/CR1/Rex	78	4842
R1/LOA/Jockey	60	3771
R2/R4/NeSL	27	1748
RTE/Bov-B	29	2891
**LTR elements**	143	10,416
BEL/Pao	43	3194
Ty1/Copia	4	346
Gypsy/DIRS1	96	6876
**DNA transposons**	376	25,824
hobo-Activator	76	6178
Tc1-IS630-Pogo	230	14,626
Other (Mirage, P-element, Transib)	4	349
**Rolling-circles**	36	2939
**Unclassified**	440	30,196
**Small RNA**	35	1999
**Satellites**	2	93
**Low complexity**	193	9749

## 2 Experimental Design, Materials and Methods

### 2.1 Sample collection and sequencing

*Elaeidobius kamerunicus* samples were captured from oil palm female inflorescence during its anthesis. All of the samples were originally collected from an oil palm plantation of PT. Gunung Sejahtera Ibu Pertiwi, Kalimantan Tengah, Indonesia, with geospatial coordinate (2°25′28.6″S 111°47′26.8″E). The samples were then identified based on their morphological characteristic. One male of *E.kamerunicus* (monoisolate) was then selected for the determination of the genome sequence.

Total genomic DNA was extracted using the gSYNC™ DNA Extraction Kit (Geneaid) following the manufacturer's instructions. The quantity and quality of genomic DNA were measured using NanoDrop spectrophotometer (Thermo Fisher Scientific) and Qubit fluorometer (Invitrogen), followed by visualization on 0.8% agarose gel.

The library for NGS was prepared using NexteraXT library prep kit, and their quality and quantity were determined using Agilent Tapestation 4200 (Agilent), Qubit fluorometer (Invitrogen), and ABI 7500 Fast System qPCR (Applied Biosystems). The library sizes of about 350–600 bp were used for sequencing. Four paired-end libraries were generated using the Illumina NextSeq 500 sequencing platform.

### 2.2 Genome assembly and evaluation

The quality of the reads was assessed with the FastQC v. 0.11.2 software [6]. Genome assembly requires the sequencing quality of each base of the read at the level of Q30 (Phred scale). The raw reads were trimmed using the Trimmomatic v. 0.17 software [7]. K-mer length estimation for genome assembly was conducted using Kmergenie software [8]. Paired and unpaired high quality reads were taken as an input to the SPAdes v. 3.10.1 genomic assembler [9] with the following options: -careful -k 17, 19, 21, 23, 25, 31, 33, 35, 37, 41, 43, 45, 47, 51, 53, 55, 57, 61. Scaffolds that were <200 bp in length were removed manually. The contaminants of foreign DNA, such as remaining adapters/vectors, organellar DNA, or contamination, were removed during submission to the NCBI GenBank database. The genome assembly statistics were obtained using QUAST software [10]. The completeness of *E.kamerunicus* genome assembly data was evaluated using BUSCO v. 3 analysis [11] against the Arthropoda database (odb9), consisting of 1066 orthologs constructed from 60 species.

### 2.3 Identification of putative simple sequence repeat (SSR)

The SSR mining data in the *E.kamerunicus* genome was performed using Krait v. 1.3.3 software [12]. Four types of genetic variation, such as perfect SSRs (pSSRs), compound SSRs (cSSRs), imperfect SSRs (iSSRs), and variable number tandem repeats (VNTRs), were analyzed.

### 2.4 Repeat identification, masking, and genome annotation

The repetitive contents detection, such as transposable elements, retroelements, and total interspersed repeats, were detected using RepeatMasker v. 4.1 [13] with default parameters and insect Dfam repeat database [14]. The repeat-masked scaffold sequences were subjected to functional gene annotation. Functional annotation and gene ontology (GO) mapping of the final set of predicted protein sequences was carried out by OmicsBox v. 1.3.11 [15], [16].

## Ethics Statement

Not applicable. No ethics protocols are required for Coleoptera in Indonesia.

## CRediT Author Statement

**Ardha Apriyanto:** Conceptualization, Methodology, Formal analysis, Investigation, Resources, Data curation, Visualization, Writing, Reviewing, and Editing; **Van Basten Tambunan:** Conceptualization, Methodology, Investigation, Resources, and Writing.

## Declaration of Competing Interest

The authors declare that they have no known competing financial interests or personal relationships which have or could be perceived to have influenced the work reported in this article.

## Appendix Supplementary Materials

Image, application 1

Image, application 2

Image, application 3

Image, application 4

Image, application 5

Image, application 6

## Footnotes

Supplementary material associated with this article can be found in the online version at doi:10.1016/j.dib.2021.106745.
